# Supplementary material for: Complexome profiling of the Chlamydomonas psb28 mutant reveals TEF5 as an early PSII assembly factor
Source: Plant Cell. 2025 Mar 17;37(6):koaf055. doi: 10.1093/plcell/koaf055 (PMC12164590; doi:10.1093/plcell/koaf055)
Supplement: koaf055_Supplementary_Data [file koaf055_supplementary_data.zip › psb28_interactive.zip!/plots/intensity/qualityPlots/peptides/_Cre11.g467350.t1.2.html]

### Cre11.g467350.t1.2

Trivial name:   
SeqCoverage [median]: 30.20  
Mass: 73.22 kDa  
  
 

| Sequence | MissedCleavage | Mass [Da] | wtIntensity | muIntensity | Score |
| --- | --- | --- | --- | --- | --- |
| AALAALSDLSGLQR | 0 | 1384.77 | 1795.81 | 0.00 | 145.28 |
| APFDVAELQR | 0 | 1144.59 | 2697.48 | 11661.31 | 155.84 |
| ELALQR | 0 | 728.42 | 0.00 | 1084.35 | 112.95 |
| ELMGLAAK | 0 | 831.45 | 438.13 | 0.00 | 100.74 |
| IIDHDNHEHR | 0 | 1284.60 | 0.00 | 500.44 | 77.38 |
| LAAAAAPALGPAQVAALAAGPQGK | 0 | 2084.17 | 1769.01 | 2620.02 | 118.37 |
| LLAAPAGGGGGGGAPSAGVAGVLR | 0 | 1932.05 | 1018.54 | 1477.22 | 81.36 |
| NEGIHGFLVR | 0 | 1140.60 | 0.00 | 2006.34 | 49.30 |
| SDTPILDYQLQQR | 0 | 1575.79 | 810.94 | 3966.74 | 94.47 |
| TVALAVGLNYVK | 0 | 1246.73 | 0.00 | 721.34 | 127.68 |
| TVALAVGLNYVKER | 1 | 1531.87 | 629.04 | 656.47 | 64.65 |
| VADQLLSGR | 0 | 957.52 | 1021.44 | 1339.41 | 125.82 |
| YNQVDNKGELFGVNV | 1 | 1694.83 | 0.00 | 3518.40 | 120.59 |
|  |  |  | 10180.4 | 29552.1 |  |
